# RETRACTED: Cao et al. Potential Biomarkers of Fatal Hypothermia Revealed by UHPLC-MS Metabolomics in Mice. *Metabolites* 2025, *15*, 116

**DOI:** 10.3390/metabo15120799

**Published:** 2025-12-17

**Authors:** Xin-Zhi Cao, Zhong-Wen Wu, Xing-Yu Ma, Wei-Liang Deng, Ding-Hao Chen, Jia-Li Liu, Jia-Hao Li, Hui Wang, Bao-Qing Pei, Dong Zhao, Qi Wang

**Affiliations:** 1 Guangzhou Key Laboratory of Forensic Multi-Omics for Precision Identification, School of Forensic Medicine, Southern Medical University, Guangzhou 510515, China; 3211009021@i.smu.edu.cn (X.-Z.C.); 3210090135@i.smu.edu.cn (Z.-W.W.); 3211009018@i.smu.edu.cn (W.-L.D.); 3210034035@i.smu.edu.cn (D.-H.C.); xing1993@smu.edu.cn (J.-L.L.); jiahao0613@smu.edu.cn (J.-H.L.); 2 Key Laboratory of Evidence Science (China University of Political Science and Law), Ministry of Education, Beijing 100088, China; 2202260347@cupl.edu.cn; 3 Department of Pediatric Surgery, Guangzhou Women and Children’s Medical Center, Guangzhou Medical University, National Children’s Medical Center for South Central Region, Guangzhou 510515, China; wanghuiwxw@gzhmu.edu.cn; 4 Beijing Key Laboratory for Design and Evaluation Technology of Advanced Implantable & Interventional Medical Devices, Beijing Advanced Innovation Center for Biomedical Engineering, School of Biological Science and Medical Engineering, Beihang University, Beijing 100191, China; pbq@buaa.edu.cn

The journal retracts the article entitled “Potential Biomarkers of Fatal Hypothermia Revealed by UHPLC-MS Metabolomics in Mice” [1], cited above.

Following publication, concerns were brought to the attention of the Editorial Office regarding errors in the metabolite identification and analysis [1].

Adhering to our complaint’s procedure, an investigation was conducted by the Editorial Office and the Editorial Board that confirmed a major methodological error that invalidates the key findings presented in Tables 1–4 and Figures 1–4. The authors fully collaborated throughout the investigation process and agreed that these errors undermine the validity of the overall findings presented in the article. Consequently, the authors and the Editorial Board have decided to retract this article [1] as per MDPI’s retraction policy (https://www.mdpi.com/ethics#_bookmark30).

This retraction was approved by the Editor-in-Chief of the journal *Metabolites*.

The authors agree with this retraction.
